# A Clinician’s Brief Guide to the Mental Capacity Act / Mental Capacity Legislation: Principles and Practice

**DOI:** 10.1192/pb.bp.113.043828

**Published:** 2014-06

**Authors:** Ben Spencer

The Mental Capacity Act 2005 is here to stay, and increasingly psychiatrists are requested to provide an opinion on an individual’s decision-making capacity by other medical practitioners or the courts. It is fair to say that the 21st-century psychiatrist needs to have an expert-level knowledge of the Act, enabling their decisions to be defended if challenged in court. There are few options available to increase one’s understanding of the law in this area. The official Code of Practice^1^ to the Mental Capacity Act offers little clarity, and certainly not at the level needed by a practising psychiatrist or other mental health professional, whereas Jones’ *Mental Capacity Act Manual*^2^ comprises fairly dense law, and more and more psychiatrists it seems are having formal legal training. Against this background, *A Clinician’s Brief Guide to the Mental Capacity Act* and *Mental Capacity Legislation: Principles and Practice* enter the fray, both broadly attending to the psychiatrist who wishes to further their understanding of the Act.

*A Clinician’s Brief Guide* is what the Code of Practice should have been: sequentially explaining the provisions of the Act, with reference to relevant case law demonstrating how these should be applied in practice. My main criticism is that the section on ‘best interests’ loses this focus, concentrating on overarching principles and mechanics rather than a legal interpretation of the statutory criteria of the assessment of ‘best interests’.

*Mental Capacity Legislation* takes a different approach, and is focused more on developing one’s own clinical skills in assessing capacity as a clinician (crucial!) and interpreting ‘best interests’, illustrated by insightful clinical vignettes. All other provisions of the Act are covered and relevant case law mentioned, although not in as much depth as in *A Clinician’s Brief Guide*.

Both books have excellent coverage of the Deprivation of Liberty Safeguards (DoLS) provisions, and are recommended to anyone who intends to be a mental health assessor for DoLS assessments. The case law in this area is rapidly developing, but the key developments are discussed and both achieve some clarity in this notoriously complex (and at times self-contradictory) area of legislation.

Perhaps the greatest strength of these two books is that they are both pitched for a range of audiences, leading the reader through the simple principles to the controversial cases, and are perhaps among the few academic books that would suit a psychiatrist at any stage in their training. Either would leave you well versed in the Act, but they are not so different as to be complimentary - one would suffice on anyone’s bookshelf. If the reader wants a superior and accessible Code of Practice but does not want to tackle cold, hard law, then go for *A Clinician’s Brief Guide*. If on the other hand you want more understanding of how to conceptualise the provisions of the Act and inform your face-to-face clinical practice, go for *Mental Capacity Legislation*.
